# Spiritual Care[Givers] Competence in Palliative Care: A Scoping Review

**DOI:** 10.3390/healthcare12111059

**Published:** 2024-05-22

**Authors:** Cristina Costeira, Ana Querido, Filipa Ventura, Hugo Loureiro, Joana Coelho, Enric Benito, Maria Nabal, Monica Dones, Marcela Specos, Carlos Laranjeira

**Affiliations:** 1 School of Health Sciences, Polytechnic University of Leiria, Campus 2, Morro do Lena, Alto do Vieiro, Apartado 4137, 2411-901 Leiria, Portugal; ana.querido@ipleiria.pt (A.Q.); joana.c.sousa@ipleiria.pt (J.C.); 2 Centre for Innovative Care and Health Technology (ciTechCare), Polytechnic University of Leiria, Campus 5, Rua das Olhalvas, 2414-016 Leiria, Portugal; 3 Health Sciences Research Unit: Nursing (UICISA: E), Nursing School of Coimbra (ESEnfC), 3004-011 Coimbra, Portugal; filipaventura@esenfc.pt (F.V.); handre.loureiro.1@gmail.com (H.L.); 4 Center for Health Technology and Services Research (CINTESIS), NursID, University of Porto, 4200-450 Porto, Portugal; 5 Forum Ibero Americano de Espiritualidad, Fundacion SECPAL, C. Sta. Isabel n 51 Centro, 28012 Madrid, Spain; benitoenric@gmail.com (E.B.); monica.dones@gmail.com (M.D.); homemarce@yahoo.com.ar (M.S.); 6 Palliative Cares Supportive Team, Hospital Universitario Arnau de Vilanova de Lleida, Universidad de Lleida, 25198 Lleida, Spain; 7 Palliative Care Hospital Support Team, The Ramón y Cajal University Hospital of Madrid, 28034 Madrid, Spain; 8 Departamento de Enfermería, Facultad de Medicina, Autonomous University of Madrid, Calle del Arzobispo Morcillo, n° 4, 28029 Madrid, Spain; 9 Institute Pallium Latin-American, Bonpland 2287, Buenos Aires 1425, Argentina; 10 Comprehensive Health Research Centre (CHRC), University of Évora, 7000-801 Évora, Portugal

**Keywords:** spiritual care competence, palliative care, education, clinical practice, review

## Abstract

To deliver spiritual care, professionals must be skilled in physical, mental, social, and spiritual care. Spiritual care competence includes knowledge, behaviors, attitudes, and skills that enable successful or efficient care. This review aims to identify the scope of competence and the specific skills, knowledge, and attitudes used in providing spiritual care to people needing palliative care, and the main challenges and facilitators. A scoping review was developed using the Joanna Briggs Institute methodology. Six databases (Web of Science; MEDLINE/Pubmed; Scopus; CINAHL; MedicLatina and SciELO) were searched in September 2023, with an update in January 2024. The resulting 30 articles were analyzed using a content analysis approach. Information was categorized into three domains: cognitive, affective, and functional (based on three personal resources: intrapersonal, interpersonal, and transpersonal). Palliative care professionals face a lack of training and insufficient preparation to deliver spiritual care. Spiritual care competence depends on professional spiritual development and experience, spiritual intelligence (cognitive), spiritual humility (affective), and having a critical and reflexive mind (functional). In the future, palliative care should seek to improve competent spiritual care. This review could help clarify the real configuration of competent spiritual care and lead to improvements in a professional’s empowerment when delivering effective spiritual care to patients and families.

## 1. Introduction

Spiritual care is a crucial component of comprehensive and value-driven care and is regarded as an indicator of high-quality care [1,2,3]. Spiritual care is a subjective and ever-changing concept that is distinct from other forms of care. It arises when professionals recognize the transcendent part of existence and attend to a patient’s perception of reality [4]. It acknowledges and reacts to the human psyche when confronted with significant life-altering occurrences (such as birth, trauma, illness, or loss) or sorrow, and can encompass the desire for purpose, self-esteem, self-expression, religious support, and possibly for rituals, prayer, sacrament, or merely for an empathetic listener. The provision of spiritual care commences by fostering empathetic connections through human interaction and adapting accordingly to meet the needs of individuals [5].

In recent decades, spiritual care has become an important target for health researchers because evidence indicates that spirituality and spiritual care have a positive influence on mental and physical health [6,7], including quality of life, hopefulness, depression and anxiety, and the ability to cope with illness [8].

In palliative care (PC), the spiritual domain has great importance and demands adjusted person-centered care, namely in end-of-life situations, when patients and families are confronted with multiple possible life choices and the inevitability of mortality [9]. To deliver thorough and appropriate care while maintaining moral and ethical principles, the spiritual aspect must be a priority, as it aids individuals in overcoming their anxieties, concerns, and distress, while also diminishing stress levels, fostering the process of recovery, and motivating patients to discover inner peace [10,11]. 

Providing holistic healthcare is only possible if the spiritual dimension is included [3], especially for people living with a serious illness, raising issues related to the meaning of life, suffering, connection, and transcendence [10]. All dimensions of a person should be considered, since all impact their symptoms and treatment. Evidence suggests that health outcomes are improved by engaging patients, family, and friends in shared decision making [11,12,13].

The World Health Organization describes spiritual care as an essential domain, although it is still one of the most neglected components of healthcare systems [14]. While PC has always included spiritual care, its provision and inclusion in clinical care are still insufficient to meet patients’ and families’ needs [15,16]. Several studies demonstrated that patients’ expectations of spiritual discussions in the healthcare setting are not being met. Caregivers highlighted barriers such as work overload; lack of training and practical experience; deficits in self-knowledge and self-awareness regarding spirituality; “lack of spiritual support in multicultural and multireligious populations, multiculturalism poses challenges to providing spiritual care” [13] (p. 1); communication barriers; lack of attention by organizational managers to the importance of holistic care; motivational fences; blocks in interprofessional collaboration; environmental conditions; late referral for PC; and uncontrolled physical symptoms [13,17,18,19,20]. The existence of multiple religious and spiritual practices leads to greater difficulties for healthcare professionals in providing effective spiritual care [21,22]. Moreover, its operationalization has divergent meanings and practices across countries [23,24]. The diversity of religious or spiritual viewpoints has been brought about by secularization, migration processes, globalization, and the emergence of new forms of spirituality and hybridity [25]. 

The importance of having caregivers who possess spiritual care competence has been highlighted in several countries and cultures [26,27,28]. According to recent studies, there is a correlation between the spiritual care competence of caregivers and their spiritual health and performance in providing spiritual care to patients and meeting their spiritual requirements [26]. Watson’s Transpersonal Caring-Healing Theory [29] highlights the caregiver’s deliberate purpose in providing care, which might potentially strengthen the caregiver’s healing presence. This theory also recognizes the healing effects of transpersonal relationships and underlines the need to deliver care holistically. According to Anandarajah [10,30] spiritual care competence integrates (a) knowledge (encompassing comprehension of spirituality and religion, the integration of spirituality and belief in patient care, and familiarity with relevant resources and literature); (b) skills (encompassing assessment and therapy; effective communication and listening; and the ability to provide compassionate presence, deliver holistic spiritual care, and navigate differences in belief); and (c) attitudes (encompassing respect, spiritual self-awareness, self-care, and a focus on spiritual well-being). 

Providing spiritual care requires trained caregivers [10]. In this sense, knowing what is expected from a caregiver could encourage managers and curriculum planners to promote, through education and training, the spiritual ability and competence of students and caregivers in delivering spiritual care [31,32,33,34,35].

The essence of spiritual care is *being* rather than simply *doing* [36], which may be transformational for both the client and the professional. Therefore, in spiritual care, it is not only care provision that holds significance, but rather the incorporation of compassion and spirituality that contribute to the provision of comprehensive care. To date, the systematizations of indicators of competent spiritual care development and evaluation are scarce. To address this gap, this review sought to identify the scope of competence; the specific skills, knowledge, and attitudes used in spiritual care of people needing PC; and the main challenges and facilitators in its provision. In response to growing interest in spiritual care, we hope to offer a comprehensive framework of what configures appropriate and competent spiritual care. 

## 2. Materials and Methods

### 2.1. Research Question

This study investigates the following:

1. What is the scope of competence of professionals providing spiritual care to people needing PC?
2. What indicators of knowledge, skills, and attitudes are needed for the delivery of spiritual care?
3. What challenges and facilitators have been linked to the provision of appropriate and competent spiritual care?

### 2.2. Study Design

This review is part of a larger study contributing to valuing spiritual care in PC through the development of Iberian guidelines for spiritual care competence [37]. In this stage, a scoping review was carried out to find answers to the research questions with the aid of a health sciences librarian. This approach aims to summarize available evidence and map knowledge about a given concept of interest [38]. The pre-established five steps were: (i) formulation of the research question; (ii) identification of relevant sources of evidence; (iii) selection of sources of evidence for inclusion; (iv) data collection/extraction; and (v) grouping, summarizing, and reporting results [39]. 

The authors developed a search strategy and protocol. The research team comprised nursing faculty members (C.C., A.Q., and C.L.), nursing researchers (F.V. and H.L.), and one occupational therapist (J.S.). Two of the authors have significant experience in providing spiritual care to people needing PC, and three are experienced researchers in PC. 

The review was conducted and reported according to the Preferred Reporting Items for Systematic Reviews and Meta-Analyses Extension for Scoping Reviews (PRISMA-ScR) [38,40]. The study protocol was registered in the Open Science Framework platform (https://osf.io/95rsg/ accessed on 10 April 2024). 

### 2.3. Eligibility Criteria

Using the JBI framework for scoping reviews [41,42,43], the team of reviewers collectively established a set of criteria for determining which records to include and exclude. They then applied these criteria to the records obtained from searches conducted on various databases and platforms. The Population/Concept/Context (PCC) framework was used to formulate the eligibility criteria, as depicted in Table 1. The publications were limited to peer-reviewed primary and secondary research published in English, Portuguese, and Spanish. There were no limitations on the choice of time or study design. This review did not include any comparative or control measures.

### 2.4. Search Strategy

In the first phase, on 19 July 2023, a limited search was conducted on Medline via PubMed. Table 2 shows the search strategy used in the MEDLINE database—using truncation and operators (Boolean OR and AND)—to analyze the subject headings and keywords in the titles and abstracts and plan a subsequent search. In the second phase, in September 2023 and an update in January 2024, a search was conducted in six databases [Web of Science; MEDLINE via EBSCO; Scopus; CINAHL via EBSCO; MedicLatina via EBSCO; Scientific Electronic Library Online (SciELO)]. The search strategies were adapted to each database. Finally, in the third phase, the reference lists of the included studies were transferred to the Rayann software^®^ (http://rayyan.qcri.org) and analyzed.

### 2.5. Study Selection

**Figure 1:**
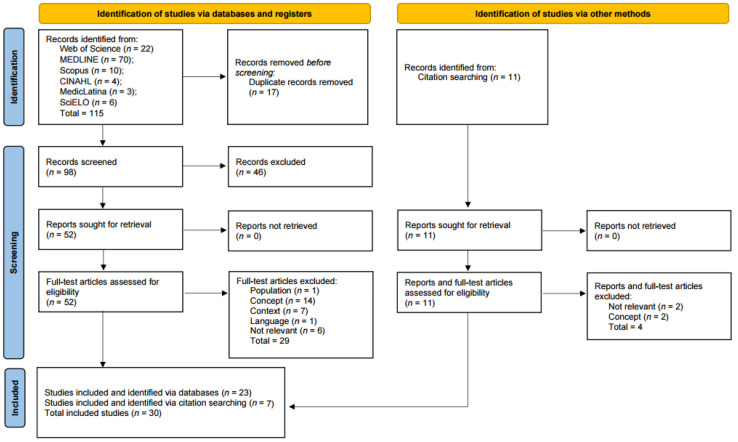
PRISMA-ScR flowchart for identifying, screening, and selecting the articles included in the scoping review.

The records (*n* = 115) were exported and uploaded to Rayyan^®^ software (Qatar Computing Research Institute, Doha, Qatar). Two independent reviewers screened the titles and abstracts, using the predetermined inclusion and exclusion criteria, and then eliminated duplicates. Eligible studies for review (*n* = 52) advanced to the second stage and were read in full by two independent reviewers, who confirmed their eligibility using the predetermined standards. The two screening phases included a standardized report on reasons for exclusion. A third reviewer intervened to resolve disagreements between the other reviewers. When there was inadequate or questionable information in the article, the corresponding author was contacted; if the author did not answer and the information requested was essential to validate the data to be retrieved, the study was not included. The total number of records detected, the reports included and excluded, the reason for exclusion, and the documents included after manually reviewing the reference lists are displayed in the PRISMA flowchart (Figure 1).

### 2.6. Data Extraction

Data were extracted with an instrument developed for this purpose by the authors, using Microsoft Excel^®^. Data were extracted and synthesized by two pairs of authors independently (C.C., H.L. and F.V., J.S.). Any disagreements between authors were discussed/analyzed with a third reviewer (J.C. or C.C. depending on the pair of researchers).

For each included study, the following data were extracted: title; authors; year of publication; country; type of study; indicators of spiritual care competence (cognitive domain, affective domain, and functional/instrumental domain); and main findings. Supplementary Materials: Table S1 summarizes the data extracted except the competence indicators presented in Table 3.

### 2.7. Data Synthesis and Reporting

A third reviewer aggregated all the extracted data into a single document. The descriptive data from the included studies were presented in tables using descriptive statistics. A qualitative content analysis was used to synthesize the textual elements, resulting in a coding structure that, through a deductive approach, led to the categorization, classification, and association of the information according to similarity and thematic affinity [44,45]. In our analysis, the deductive coding was derived from the Spiritual Care Competence Scale^®^ [SCCS] [46], the EPICC Spiritual Care Competency Self-Assessment Tool^®^ [5,47], and the spiritual support model proposed by the spirituality working group of the Spanish Palliative Care Society (SECPAL) [48]. In this sense, this review used the three domains of spiritual care competence: (a) cognitive; (b) affective; and (c) functional (based on three resources: intrapersonal, interpersonal, and transpersonal).

**Table 3:** Indicators of spiritual care competence and the studies in which they were identified.

Cognitive (knowledge)—Assessing and planning spiritual care [5,47]	**Intrapersonal Resources [48]**	**Interpersonal Resources [48]**	**Transpersonal Resources [48]**
	Understanding the concept of spiritual care [5,27,36,47,49,50,51,52,53,54,55,56,57,58,59,60,61,62,63,64,65]	Awareness of different approaches to spiritual assessment [21,27,36,47,50,51,52,53,54,55,59,61,62,65,66,67,68]	Integration of being human into the evolutionary axis of its existence [5,21,46,47,50,53,58,59]
	Understanding the impact of personal values and beliefs in providing spiritual care [5,21,36,47,51,52,53,59,68,69]	Understanding other professionals’ roles in providing spiritual care [36,47,53,61,63,67]	Problem-solving ‘solution-seeking’ through the caring process of knowing/being/doing/becoming) [5,21,46,47,49,50,51,53,61,63,64,65]
	Explaining, to oneself and others, the impact of spirituality on health and well-being across one’s lifespan [5,21,26,36,47,59,63,65,68,70]	Understanding the concept of compassion and presence and its importance in spiritual care [5,46,47,53,54,63,67]	
		Knowing how to respond appropriately to identified spiritual needs and resources [21,36,47,50,51,53,54,55,59,61,62,63,65,69]	
		Knowing how to evaluate whether spiritual needs have been met [47,51,52,53,54,55,58,59,61,63]	
		Understanding the ways that people express their spirituality [26,36,47,49,51,53,55,61,65]	
		Awareness of the different world/religious views and how these may impact people’s responses to key life events [5,36,46,47,49,50,51,53,54,55,61]	
Affective (attitudes)—Self-assessment, personal support and patient counseling-, attitude towards patient spirituality [5,47]	**Intrapersonal Resources [48]**	**Interpersonal Resources [48]**	**Transpersonal Resources [48]**
	Willing to explore personal, religious, and spiritual beliefs [21,26,47,50,52,57,64,68,70]	Being trustworthy and respectful of people’s expressions of spirituality and different world/religious views [5,47,49,50,51,53,59,61,62,63,64,65,71,72]	Developing and sustaining loving, trusting, and caring relationships [5,63]
	Respecting and being open to people’s diverse expressions of spirituality [47,49,50,51,53,55,59,61,64,73]	Being open, approachable, and non-judgmental [5,21,46,47,49,50,53,55,59,61,62,63,65,66,72]	Creating a healing environment at all levels; a subtle environment for energetic, authentic, and caring presence [21,46,47,48,49,50,51,53,54,57,58,59,61,63]
		Being compassionate and being present [21,47,49,50,53,54,59,61,63,67]	Being open to spiritual, mysterious unknowns; allowing for miracles [5,21,46,47,48,50,57,58,63]
		Willing to deal with emotions [47,50,53,61,63,64]	
		Willing to collaborate with and refer to others (professionals/non-professionals) when providing spiritual care [5,46,47,50,53,61,63,67]	
		Welcoming, accepting, and showing empathy, openness, professional humility, and trustworthiness when seeking additional spiritual support [47,49,50,53,54,59,61,63,64,65,67]	
Functional/instrumental (skills)—Intervention and evaluation of spiritual care/communication strategies used to support [5,47]	**Intrapersonal Resources [48]**	**Interpersonal Resources [48]**	**Transpersonal Resources [48]**
	Reflect meaningfully upon own values and beliefs and recognize these may be different from other people’s [47,53,59,61,68]	Recognizing the uniqueness of people’s spirituality [47,49,50,51,53,57,59,60,61,63,64,68]	Develop transpersonal experiences such as mindfulness, yoga, visualization exercises, mental relaxation, making mandalas, body awareness activities, therapeutic writing, etc. [21,48,50,54,56,60,61,63,66]
	Taking care of personal well-being [5,26,47,48,52,60,64,68,70]	Interacting with and responding sensitively to people’s spiritual diversity [21,36,47,49,50,51,53,55,58,59,61,63,65,67,73,74]	Develop interventions to sustain human dignity [5,21,30,36,46,47,48,49,50,51,52,53,54,55,56,57,58,60,61,62,63,64,65,66,67,68,69,70,71,72,73,74]
	Demonstrate spiritual self-awareness [48,53,66,73]	Listening skills (verbal and non-verbal practices) [5,21,36,46,47,48,49,50,53,54,58,59,61,63,64,67]	
		Group communication, conflict resolution, negotiation, and goal clarification [36,48,49,53,61,64]	
		Conducting and documenting a spiritual assessment to identify spiritual needs and resources [5,46,47,48,49,50,51,53,54,56,58,61,62,63,64,66,71,73]	
		Collaborating with other professionals in the provision of spiritual care [21,47,50,53,67]	
		Containing and dealing appropriately with emotions [21,47,50,53,61,67,74]	
		Recognizing personal limitations in spiritual caregiving and resorting to others when appropriate [21,46,50,62,67,68,72,73]	
		Using evidence-informed practices to help patients and families address fears and spiritual and other distress related to life-limiting and end-of-life care [5,46,47,48,50,53,55,58,61]	
		Applying culturally appropriate, evidence-informed strategies for communicating with patients and families about pain and suffering, loss, complicated and anticipatory grief, and life review [5,46,47,48,49,50,51,53,55,59,61,63]	
		Evaluating and documenting personal, professional, and organizational aspects of spiritual care, and reassessing appropriately [47,49,51,53,61,65]	

The included studies were not submitted to critical appraisal because the goal of this type of study is to identify gaps in the literature and propose potential research questions for systematic reviews [45].

## 3. Results

The initial search retrieved 115 articles. After removing 17 duplicates, the remaining 98 articles were reviewed by title and abstract, and 46 articles were excluded. The full review was performed on 52 articles. Among these, 29 were excluded because they did not align with the study criteria. Eight additional articles were retrieved by searching article bibliographies. In the end, a total of 30 articles were included in the scoping review. The results of the search are shown in a flow diagram (Figure 1). 

**Figure 2:**
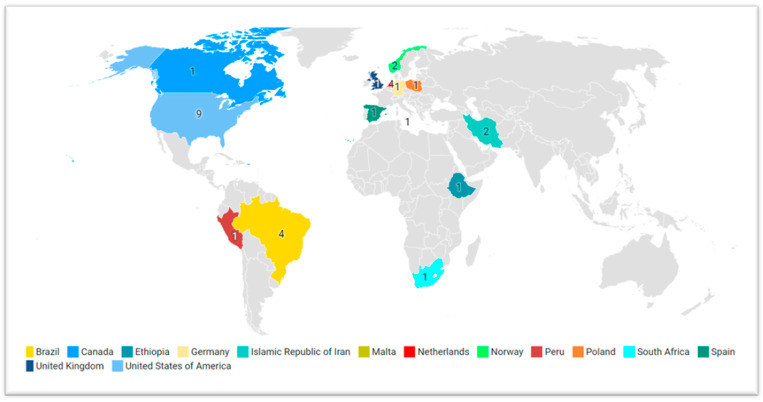
Geographic distributions of studies included in the scoping review.

All retrieved studies were published between 2007 and 2022. Most of the articles were primary studies (*n* = 19), published in 2022 (*n* = 7), and written in English (*n* = 25). Studies were developed with health professionals delivering PC in different clinical contexts (*n* = 19), education/formation in PC (*n* = 6), and specialized PC services (*n* = 5). The country that contributed the largest number of studies was the United States of America (*n* = 9) (Figure 2).

The data extracted from the included studies were categorized into three domain categories: cognitive, affective, and functional. 

In the cognitive domain, the imperative lies in the assimilation of knowledge that helps professionals formulate a thorough spiritual assessment and tailor a customized spiritual care plan. In the affective domain, professionals need to show a predisposition towards executing proficient spiritual care and be equipped with the necessary tools to achieve personal emotional balance, thereby ensuring the delivery of spiritual care that satisfies the needs of the patient and their family. In the functional domain, professionals must demonstrate competencies that empower them to lead supportive interventions and conduct evaluations of effective spiritual care within the clinical practice context (Table 3).

For each of these domains, the professional should engage intrapersonal, interpersonal, and transpersonal resources. This not only enables them to deliver proficient care to patients and their families but also fosters the development of their spiritual well-being.

The results presented in the subsequent sections were organized according to the cognitive, affective, and functional domains. This organization is rooted in the analysis of data extracted from the articles included in the review, with each domain serving as a framework for categorizing and discussing the extracted information. 

### 3.1. Cognitive Domain

In the cognitive domain, professionals are expected to acquire and retain knowledge that enables them to comprehend, articulate, and be aware of the nuances of spiritual care [47]. 

In the realm of intrapersonal resources, professionals are expected to leverage their knowledge to recognize the importance of spirituality in influencing health and well-being. There is consensus in the reviewed literature about the criticality of comprehending spiritual care, acknowledging the variability across different cultures and individuals, and elucidating the profound effects that these variations have on health and well-being throughout the human lifespan. The studies underscore the multiplicity of spirituality definitions, which are inherently subjective and shaped by an individual’s age, experience, and cultural background [26,27,49,50,51,53,59,61]. This diversity complicates the establishment of a universally accepted spiritual care competence and interferes with expected and delivered spiritual care. The ability to differentiate between spirituality and religion is deemed crucial [54,60] to understand and articulate how personal spiritual experience influences the delivery of spiritual care [21,26,51,52,53,59,68,69]. This skill extends to defining key concepts such as PC, religion, spirituality, and spiritual care itself, as suggested by Dezorzi et al. [55], thereby enriching the professional’s capacity to provide nuanced and culturally sensitive spiritual care.

In the cognitive domain, interpersonal resources pertain to proficiency in engaging with the person’s spiritual dimension, while acknowledging the diversity of spiritual and cultural worldviews, beliefs, and practices [47]. Professionals must take various approaches to spiritual care, comprehend the roles of other professionals in this realm, and grasp the significance of compassion and presence in the provision of spiritual care. Professionals should be capable of appropriately identifying spiritual needs and resources, assessing the fulfillment of these needs, understanding the diverse expressions of spirituality, and considering how the varying global and religious perspectives might influence individual responses to significant life events [50]. 

Furthermore, professionals are tasked with discerning whether patients have a restful form of religiosity that is deeply integrated and supportive during health challenges, or if conflicts within their belief system may arise [52]. The availability of instruments to assess the dimensions of spiritual care is crucial in enabling professionals to devise a targeted plan to address spiritual care needs effectively, thereby facilitating competent spiritual care delivery [27,48,49,50,51,52,55,68].

Lastly, professionals with transpersonal resources are expected to have the capability to assess and address spiritual needs and resources, fostering the integration of the human experience within a broader evolutionary context, and thus cultivating an awareness conducive to problem-solving and the provision of compassionate care [36,50,61]. It is important to acknowledge that human existence is intrinsically linked to the individual and communal pursuit of meaning, purpose, and transcendence [50]. This pursuit is characterized by how individuals connect with the present moment, themselves, others, nature, and the significant or sacred realms, thereby underscoring the holistic nature of human life [36,50,51,61]. 

### 3.2. Affective Domain (Attitudes, Behaviors)

The affective domain encompasses the emotional aspects of spirituality, focusing on the attitudes and behaviors that influence the interactions between professionals and patients. Within this domain, it is crucial to cultivate a trusting therapeutic relationship, which is foundational to effective practice. A compassionate disposition, characterized by empathy and a genuine concern for the well-being of others, involving passion and love, is essential in this domain [50,60]. Such compassion transcends mere sympathy, inviting a deep engagement with the patient’s experience of suffering, thereby enabling healthcare professionals to provide care that is not only empathetic and devoid of pity but also deeply attuned to the needs of patients and their families [50].

In this domain, it is critical to integrate ethical reasoning: respecting a client’s decisions about their well-being; avoiding judgmental attitudes; being a reflective caregiver; sustaining the ability to contain/tolerate ambiguity; developing a tolerance for sadness capable of empathizing with the suffering of others; exhibiting spiritual humility; and realizing that it is not easy to answer all questions and that quick solutions are not always possible, demanding a mutual search of meaning [21,53,61].

A person’s capacity for ethical thinking must be developed, as human nature strongly leans toward egotism, prejudice, self-justification, and self-deception [61,75]. Sometimes people confuse ethics with behaving according to social conventions, religious beliefs, and the law, but ethics is a domain unto itself, with universal principles and concepts that are transcultural and trans-religious [61,75].

In the affective domain, the professional’s intrapersonal resources pertain to cultivating mechanisms and tools for self-exploration to deepen their understanding of their personal beliefs and values [52]. This introspection is critical for discerning what holds significance for them, thereby enhancing their capacity to deliver spiritual care that is both pertinent and efficacious [52]. Moreover, the ability to reflect on one’s attitudes and approaches to various situations fosters a heightened awareness of one’s values, biases, and emotional responses. Such self-awareness is instrumental in recognizing the complex interplay between the feelings, beliefs, and values manifested in interactions with others, which is essential for the provision of effective spiritual care [50]. 

Professionals are expected to provide open and respectful spiritual care that acknowledges the diverse expressions of spirituality among individuals (i.e., interpersonal resources). Benito et al. [50] describe a model wherein attitudes of hospitality, presence, and compassion help patients and their families in their spiritual awakening. In this model, hospitality refers to the ability that professionals should develop to break the narrowness of their fears and allow a stranger in. Presence implies being deeply there for the patient and their family. Compassion is the attitude of demonstrating a truly active interest in a patient’s suffering, showing a continuing determination to do everything possible to relieve their suffering [50].

This demands a personal and professional development of empathy and compassion [54]; approachability and presence [55]; kindness and attentiveness [52]; sensitiveness, a non-judgmental posture, a reflective practice, an ability to manage ambiguity and tolerate sadness and courage when facing the suffering of others, humbleness [53], and trustworthiness [53,59].

Transpersonal resources highlight the importance of recognizing the interconnection between physical, emotional, and spiritual dimensions. The emphasis relies on the significant roles that faith and hope play in people’s lives, especially in the face of life’s uncertainties and challenges, such as illness, pain, stress, despair, sadness, fear, and death [21]. The deeply spiritual nature that characterizes us—as beings interconnected with ourselves, others, and the universe—is a dynamic experienced on a transrational, transpersonal, and transconfessional level [50].

### 3.3. Functional Domain (Skills)

The functional domain concerns the practical aspects of spiritual care, including the implementation of interventions and the effective evaluation of such care in clinical practice. This domain is critical for demonstrating competence in clinical care provision, which includes assessment, planning, intervention, and evaluation.

Professionals must manifest spiritual self-awareness, encompassing an understanding of both their personal values and beliefs and those of others, while also ensuring their well-being (i.e., intrapersonal resources). This self-awareness enhances the ability to provide competent spiritual care, drawing on personal knowledge and both own and observed spiritual experiences [73]. Personal well-being serves as a fundamental element in PC settings, where professionals frequently encounter challenging scenarios. Therefore, the development of strategies aimed at enhancing personal well-being and reframing emotionally charged experiences, such as spiritual distress among terminally ill patients, is imperative [70].

Interpersonal resources involve the adoption of behaviors that respect the uniqueness of people’s spirituality, aligned with an interaction with sensibility. When professionals interact and respond to patients, sharing their vulnerability and their transcendental experience, they help patients cross the bridge from suffering to acceptance and surrender, towards transcendence and with it to “spiritual healing”, which refers to the person’s ability to find solace, comfort, connection, meaning, and purpose amid suffering, heartbreak and, pain [50]. Through compassionate interventions, the sufferer transcends to a different space characterized by growth and a more mature vision of reality [50].

These resources encompass the use of proficient communication strategies to facilitate the provision of effective spiritual care. From this perspective, professionals are expected to engage with patients and their families through assertive communication regarding spiritual or religious matters, characterized by both acceptance and sensitivity. Professionals must establish objectives that align with the spiritual or religious perspectives of patients. Furthermore, this requires adapting therapeutic approaches to incorporate the spiritual or religious viewpoints of patients, based on evidence [49]. 

The reviewed literature acknowledges the importance of recognizing personal limitations in spiritual care and refers to multidisciplinary professionals, since spiritual competence is developed differently among professionals and not all are prepared to deliver it [50,61].

In the domain of transpersonal resources, professionals are anticipated to foster and engage in transpersonal experiences that uphold human dignity and respect the autonomy of patients. The research underscores the significance of incorporating self-reflective practices such as journaling, prayer, meditation, and artistic endeavors [36,50]. These activities reflect the commitment to explore the emotions, beliefs, and values of others, thereby facilitating personal and communal growth. Activities may include engaging in prayer, studying spiritual texts like the Bible, practicing active listening, providing comforting verbal reassurances, ensuring a consistent and supportive presence, coordinating visits from spiritual leaders, and personalizing care with gestures like nail painting for special occasions. Additionally, the introduction of therapeutic interventions such as music, massage, therapeutic touch, and mindfulness practices are highlighted [21,54,61,66]. Such comprehensive care, which attends to both basic and spiritual needs, establishes a healing environment that encompasses both physical and metaphysical elements, thereby exemplifying proficient spiritual care within this domain [21].

### 3.4. Novice–Expert Continuum

The capacity to provide tailored interventions that suit each client and enhance their sense of self-worth is a key aspect of spiritual care competence, which is an active and continuous process. Spiritual competence is situated on a continuum extending from spiritually negative to spiritually competent practice, which requires reflection on experience. This continuum is similar to Benner’s [76] continuum from novice to expert. A collection of abilities, attitudes, and knowledge that may be acquired via practice and education throughout time defines this competence continuum. For Baldacchino [77], professionals providing spiritual care should exhibit characteristics such as (a) role modeling for junior caregivers; (b) education on spirituality, integrated into undergraduate and postgraduate course programs; (c) reflection in and on the action; (d) vocation, or responding to a spiritual call; (e) taking initiative for active presence in care; and (f) commitment towards the delivery of spiritual care. To achieve the adage of “Being in Doing”, spiritual humility, spiritual intelligence, reflection, and critical incident analysis are indicators of competence from novice to expert [36]. Humility as a “teacher of all virtues” impacts the relational functioning of dyads, groups, and communities [50,53,78]. It has been associated with generosity, empathy, quality social relationships, spiritual maturity, and graciousness in receiving from others [78]. Wright et al. [79] asserted that the core of humility should best be described as “a particular psychological positioning of oneself—namely, one that is both epistemically and ethically aligned.” Both intrapersonal and interpersonal dimensions of humility allow professionals to (a) cultivate healthy and mature relationships and (b) develop mature forms of “alterity” or socially just and mature ways of relating across human differences. 

Furthermore, learning via reflection while acting is crucial since it enables one to assess one’s actions, enhance patient care, and acquire the essential skills [80,81]. Metacognition skills are crucial for spiritual intelligence because they enable the identification of self-actualizing wants and objectives and help direct one’s personal efforts toward these objectives. Professionals who possess metacognition are also better able to learn from their experiences, become conscious of their thinking, and have a firm understanding of who they really are—all of which are essential components of a reflective mind. The goal of both spiritual intelligence and metacognition is to fulfill an individual’s ability to increase their knowledge of their existence, including psychological components like self-perception, self-experience, and self-control [61,82]. 

### 3.5. Challenges and Facilitators Associated with the Provision of Appropriate and Competent Spiritual Care

The included studies predominantly indicate a lack of training concerning the integration of spiritual and religious considerations within the cognitive, affective, and functional domains of care. This is particularly evident in the context of developing the ability to discern when and how to engage with patients in a supportive manner (even under challenging circumstances, such as delusional states or crises), thus underscoring the imperative for clear guidelines [52,65]. Another challenge includes the lack of knowledge related to religion and the other deeply held beliefs of various people groups. Likewise, spiritual self-analysis may be a struggle, as staff can feel inadequate in delivering spiritual care and interdisciplinary communication may be inconsistent [83].

To address this shortfall, ongoing education is deemed critical for equipping PC professionals with the requisite skills to implement a holistic and person-centered care model that prioritizes the patient–family unit and incorporates spiritual care needs into the decision-making process and bereavement support [55,74]. Compassion and empathy are core values in delivering high-quality, person-centered care. These essential values can be fostered through various reflective practices, including individual and group reflection, case discussions, written exercises, debriefing sessions, simulation activities, role-playing, and shadowing other experienced professionals in the multidisciplinary team, thereby enhancing professionals’ proficiency in compassionate engagement and spiritual level [54,62,64]. It is recognized that professionals must proactively pursue personal and experiential learning opportunities to update and expand their spiritual knowledge base [60]. Evidence also suggests that ‘spiritual intelligence’ can affect the spiritual care competence of students by promoting a high level of critical thinking and spiritual self-awareness [84].

Furthermore, the development of competencies in spiritual care is highlighted as a crucial aspect of training programs [68]. The acquisition of such competencies relies on diverse pedagogical methods, depending on the individual’s spiritual awareness and experiential learning. Some strategies such as self-reflection or journaling, reflection in small groups, and managing spiritual or religious conversations are opportunities for spiritual care training. Involving a spiritual assistant in simulated learning was also well received [36,83].

Mächler et al. [69] emphasized the interrelationship between an individual’s spirituality and their professional conduct and their competencies in spiritual care, which significantly influences both patient outcomes and professional development [69]. This interplay demands careful consideration by healthcare administrators and PC professionals when structuring care delivery, organizing the workplace, and creating conducive environments for the practice of spiritual care [71]. 

The significance of a supportive environment in the provision of spiritual care is also highlighted, particularly in ensuring privacy and confidentiality during sensitive discussions (i.e., room or place where others can overhear the conversation), which extends beyond structural or organizational dimensions [52].

## 4. Discussion

The main purpose of this study was to scope the competence of professionals providing spiritual care to people experiencing PC needs. In this regard, it is important to differentiate two interchangeably usable concepts: competence and competency. Competence is the ability to do something successfully or efficiently and is a state of being prepared to do a job. In contrast, competency is usually described as an action, focusing on an individual’s actual performance in a particular situation (i.e., competence is what we do, and competency is how well we do it) [85]. 

This review proposes three important domains of spiritual care competence: cognitive and spiritual intelligence; spiritual humility; and effective and critical reflection, where the professional shows the ability to learn with continuous critical reflection through spiritual experiences. These three domains must be present for spiritual competence and are seen as a process of permanent development, with different levels until one becomes an expert. To attain competence as an expert demands that professionals have high cognitive ability (i.e., spiritual intelligence), a favorable attitude towards spiritual issues (i.e., spiritual humility), and a critical functional domain (i.e., reflective mind and metacognition) that could help them use spiritual intelligence in everyday problem-solving and efficiently satisfy the spiritual needs of patients [17,78,86,87].

As spiritual beings, we all need spiritual care in our life path. This is more evident in end-of-life situations or circumstances that induce reflection about life’s meaning, as frequently occurs in those needing PC [50,61,88]. Despite this, professionals consider spiritual care as an important but neglected aspect of healthcare [48,54,88]. Although spiritual accompaniment should be provided to all patients, namely those in the process of dying, not all professionals can do it efficiently. Spiritual care requires more than the accumulation of knowledge, demanding one be spiritual in personal and professional life [50]. This allows increased self-awareness, empathy for the client’s perspective, and the capacity to carry out tailored interventions that are suitable for each client [36]. 

This process begins by recognizing the importance of spiritual training to personal and professional spiritual development. There is a lack of professional training and a non-consensual structure in the curriculum of healthcare professionals, although studies show that educational and training programs are efficient strategies for developing skills and promoting competent spiritual care [36,67,89,90,91]. Techniques like role-playing, focus groups, reflective writing, mentorship programs, discussions on the observed delivery of holistic care, the use of art to express complex spiritual care concepts, involvement in research, tutorials, role-modeling, experiential learning, and community visits could also be beneficial in developing competence and learning in spiritual care [36,92]. Therefore, it is recommended that all PC training and curriculum incorporate spiritual care activities, because these enable caregivers to grow spiritually and better help others in need of spiritual guidance [93,94]. 

The lack of knowledge about spiritual care limits competent spiritual care [95]. It is also known that the knowledge obtained through continuous training can help professionals blossom their spirituality. The studies in this review suggest that professionals with more self-awareness of spirituality were more well prepared to deliver competent spiritual care [53,66,73]. Knowing more and the best of oneself is essential to align one’s work with the PC philosophy and find a balance between giving and receiving [96]. However, training and education are not the only aspects that interfere with spiritual development and spiritual care competence.

A diversity of personal and spiritual conceptions, experiences, and personal characteristics (i.e., age, and cultural belonging) compete for different approaches and priorities in spiritual care delivery. Spirituality is related to culture and plays a vital role in the treatment provided to patients [60]. Variables such as age and spiritual care training were significantly associated with competent spiritual care [26,27]. Lópes-Tarrida et al. [97] agree with that idea and add gender as relevant to how spiritual care is delivered. The authors defend that women are more proficient in distinguishing between spirituality and religion and more self-aware of other’s needs [97]. This aspect could be explained by education and traditional gender differences, depending on different aspects of the environment (contextual); on patients’ beliefs, experience, and current conceptions; and on family issues, professional experience, personality, knowledge, skills, and attitudes. All these will affect the process of delivering spiritual care and how spiritual care competence is conceptualized.

Competent spiritual care must involve the cognitive, affective, and functional domains, which are interconnected and mutually influence each other, encompassing one unique competence in spiritual care. Although the instruments to assess spiritual care competence defend the existence of several competencies, this review highlights the idea that these three domains must be developed to provide competent spiritual care [46,47,48,50]. The domains are not necessarily on the same level and allow the development of specific skills, values, attitudes, beliefs, behaviors, and knowledge, through the mobilization and development of three main resources: intrapersonal, interpersonal, and transpersonal specificities. The idea is based on the assumption that PC professionals must develop those domains (over several stages) to competently respond to the spiritual needs of the patient/family, suggesting an active, dynamic, and continuous process, enhanced and deepened with experience and training. 

This process is an indicator of competent spiritual care and the professional’s ability to promote person/family-centered care. It is well known that a person-centered practice brings a positive impact, since it promotes sustainable healthcare systems and high care quality [98]. With competent spiritual care, patients and families experience a sense of “healing” where they can find comfort, security, meaning-making, and closure in PC [50,99]. Thus, delivering competent spiritual care is an integral part of PC, where supporting relationships should be a central focus [50,100]. Spiritual care is characterized by the provision of a healing presence, therapeutic use of self, intuitive sense, spiritual viewpoint exploration, person-centeredness, meaning-centered therapy intervention, and the development of a spiritually nourishing atmosphere [4,50].

### 4.1. Strengths and Limitations 

One of this review’s merits is the adoption of thorough, open procedures that were followed throughout. The methodology was examined by a research team with experience in scoping reviews and PC knowledge. Six electronic bibliographic databases were searched, together with the snowball method for further research and reports, thus guaranteeing a thorough search of the literature. Furthermore, the models that support the deductive analysis were based on an anthropologic and transconfessional spiritual care approach. This means we welcomed different cosmovisions rooted in our primordial awareness of the human spirit. 

Apart from the limitations inherent to the chosen method, this literature review was also limited by its biased cultural representation among the included studies, providing a perspective of spiritual care competence focused on the occidental worldview. This could limit a full understanding of spiritual care competence in a world that is increasingly global. Most studies were carried out in Western nations, focusing on groups that have similar characteristics and come from a largely Judeo-Christian background. Furthermore, the task of clearly defining the boundaries between the ideas of religion, spirituality, and spiritual care activities is challenging due to their tight interconnection. Certain detailed information may have been obscured throughout the analysis. While we conducted extensive searches in several prominent health databases, we did not include certain databases that focus on sociological and theological studies. Lastly, findings should be regarded with caution due to the methodological and contextual differences across the studies included. These differences presented issues when analyzing, summarizing, and discussing the findings.

### 4.2. Practical Implications

Based on this review, the factors related to the development of spiritual care competence should be a concern, namely in PC. In this context, spirituality is paramount for all involved and plays an important role in minimizing suffering. Professionals are challenged to answer patient and family needs in spiritual care, and they are also confronted with their own spiritual needs, which should be satisfied and respected. Professionals recognize their own need for spiritual development to grow professionally and personally. Self-care and self-awareness are some of the main targets to help professionals deal effectively with the demanding needs of patients, families, and other team elements. If professionals are not comfortable with spiritual issues, they will not be able to mobilize and add the available efficient tools in spiritual care, aggravating the suffering of patients, families, and professionals.

Implementing strategies (such as structured educational and training programs) that could develop professionals’ spiritual awareness, spiritual knowledge, and spiritual attitude will contribute to improving professionals’ spiritual intelligence, spiritual humility, and continuous critical reflection on personal and professional spiritual experiences. This investment should be made in undergraduate and postgraduate education to improve the quality of students’ performances in delivering spiritual care to patients and families, by adopting an eclectic approach that embraces diversity within society [101].

Research in this area should develop instruments that assess spiritual competence as a specific competence, a sum of knowledge, skills, attitudes, and behaviors (i.e., the cognitive, affective, and functional domains). The findings of this review indicate the need for additional comprehensive research to evaluate the effectiveness of spiritual care interventions in enhancing the outcomes of patients, families, and clinicians.

## 5. Conclusions

This review aimed to identify the scope of competence and the specific skills, knowledge, and attitudes used in providing spiritual care to people needing palliative care, and the main challenges and facilitators through an evidence mapping method. Providing competent spiritual care is a right of patients and families in PC and a duty of professionals. To ensure this, all stakeholders in PC should be aware and truly involved. Patients/families should require quality spiritual care and professionals should understand the importance of developing spiritual care competence in the cognitive, affective, and functional domains. 

Furthermore, PC professionals must develop and mobilize intrapersonal, interpersonal, and transpersonal resources to promote real competent spiritual care based on a person-centered approach. Promoting spiritual self-awareness and increasing spiritual maturity, through a reflective mind, are the strongest predictors of effective spiritual care. Spiritual self-awareness is achieved by engaging in life events, pursuing education, and practicing critical reflection. These activities contribute to enhancing existential and spiritual well-being, while also raising one’s consciousness to a more elevated state. The process of attaining a higher level of awareness involves establishing connections with oneself and seeing patterns that enable one to develop a heightened sensitivity to the spiritual needs of others. Therefore, health managers should be aware of the impact of spiritual care investment on the health and well-being of patients and professionals. Investment in this area should be a political concern.

## Figures and Tables

**Table 1:** Eligibility criteria.

PCC Framework	Inclusion Criteria	Exclusion Criteria
Population	All papers with reference to spiritual care competence of PC professionals	All papers about healthcare recipients (e.g., patients and families)
Concept	All papers exploring spiritual care competencies (knowledge, skills, and attitudes)	Papers that discuss related sub-elements of spirituality (such as connectedness) with no reference to spiritual care
Context	All papers focusing on spiritual care education and/or practice	All papers without reference to PC
Format	Primary studies (quantitative, qualitative, and mixed methods); literature reviews; and reports, guidelines, and other technical publications by professional regulatory agencies, professional groups, scientific societies, or other organizations that have acknowledged authority and standing in the field of PC	Editor letters; opinion papers; editorials

**Table 2:** Search strategy used in Medline via PubMed.

	Title-Abs-Key
	(MH “Nurs *”) OR (MH “Health Personnel”) OR (MH “Chaplain *”) OR (MH “Psycholog *”) OR (MH “Social Worker *”) OR (MH “Counselor *”) OR (MH “Physical Therapist *”) OR (MH “Occupational Therapist *”) OR (Carer *) OR “Spiritual assistant” OR (MH “Caregiver *”) OR (MH “Palliative Medicine”)
AND	“Spiritual care competence” OR (MH “Spirituality”) OR “Spiritual learning outcomes” OR “Spiritual training skills” OR (meaning (life OR death)) OR “faith” OR religi *
AND	(MH “Palliative Care”) OR (MH “Hospice and Palliative Care Nursing”) OR (MH “Palliative Medicine”) OR “end of life” OR (MH “Hospice Care”) OR (MH “Hospices”) OR “community end of life” OR “palliative assistance” OR “last days and hour of life” OR (MH “Terminal Care”) OR (MH “Ambulatory Care”) OR “limited life” OR palliati * OR hospice * OR dying

MH—MeSH terms; * truncation.

## Data Availability

All data generated or analyzed during this study are included in this article. This article is based on the first author’s master’s dissertation in Palliative Care at the School of Health Sciences—Polytechnic University of Leiria.

## Supplementary Materials

The following supporting information can be downloaded at: https://www.mdpi.com/article/10.3390/healthcare12111059/s1, Table S1: Summary of the included studies; File S2: PRISMA-ScR Checklist.

## References

[1] Balboni T.A., VanderWeele T.J., Doan-Soares S.D., Long K.N.G., Ferrell B.R., Fitchett G., Koenig H.G., Bain P.A., Puchalski C., Steinhauser K.E. (2022). Spirituality in Serious Illness and Health. JAMA.

[2] Ghorbani M., Mohammadi E., Aghabozorgi R., Ramezani M. (2021). Spiritual care interventions in nursing: An integrative literature review. Support. Care Cancer.

[3] Tavares A.P., Martins H., Pinto S., Caldeira S., Pontífice Sousa P., Rodgers B. (2022). Spiritual comfort, spiritual support, and spiritual care: A simultaneous concept analysis. Nurs. Forum.

[4] Ramezani M., Ahmadi F., Mohammadi E., Kazemnejad A. (2014). Spiritual care in nursing: A concept analysis. Int. Nurs. Rev..

[5] van Leeuwen R., Attard J., Ross L., Boughey A., Giske T., Kleiven T., Mcsherry W. (2021). The development of a consensus-based spiritual care education standard for undergraduate nursing and midwifery students: An educational mixed methods study. J. Adv. Nurs..

[6] Özakar Akça S., Gülnar E., Özveren H. (2022). Spiritual Care Competence of Nurses. J. Contin. Educ. Nurs..

[7] The Lancet Regional Health-Europe (2023). Time to integrate spiritual needs in health care. Lancet Reg. Health Eur..

[8] Puchalski C.M., King S.D.W., Ferrell B.R. (2018). Spiritual Considerations. Hematol. Oncol. Clin. N. Am..

[9] Lewicki A. (2023). Spiritual Care. Understanding End of Life Practices: Perspectives on Communication, Religion and Culture.

[10] Salas V.C., Taboada R.P. (2019). Espiritualidad en medicina: Análisis de la justificación ética en Puchalski. Rev. Med. Chil..

[11] Sajja A., Puchalski C. (2017). Healing in modern medicine. Ann. Palliat. Med..

[12] Puchalski C.M., Cobb M., Puchalski C., Rumbold B. (2012). Restorative Medicine. Spirituality in Healthcare.

[13] Matos J., Querido A., Laranjeira C. (2024). Spiritual Care through the Lens of Portuguese Palliative Care Professionals: A Qualitative Thematic Analysis. Behav. Sci..

[14] Quinn B., Connolly M. (2023). Spirituality in palliative care. BMC Palliat. Care.

[15] Best M.C., Vivat B., Gijsberts M.J. (2023). Spiritual Care in Palliative Care. Religions.

[16] Vivat B., Lodwick R., Merino M.T.G.B., Young T. (2023). What Do Palliative Care Professionals Understand as Spiritual Care? Findings from an EAPC Survey. Religions.

[17] Guo W., Liu X., Zhang Y., Chen R., Qi W., Deng J., Cui J. (2023). Competence and perceptions of spiritual care among clinical nurses: A multicentre cross-sectional study. J. Clin. Nurs..

[18] Kuckel D.P., Jones A.L., Smith D.K. (2022). The Spiritual Assessment. Am. Fam. Physician.

[19] Laranjeira C., Dixe M.A., Querido A. (2023). Perceived Barriers to Providing Spiritual Care in Palliative Care among Professionals: A Portuguese Cross-Sectional Study. Int. J. Environ. Res. Public Health.

[20] Momeni G., Hashemi M.S., Hemati Z. (2022). Barriers to Providing Spiritual Care from a Nurses’ Perspective: A Content Analysis Study. Iran J. Nurs. Midwifery Res..

[21] Evangelista C.B., Lopes M.E.L., Costa S.F.G.d., Batista P.S.d.S., Duarte M.C.S., Morais G.S.d.N., de Sá França J.R.F., da Mata Ribeiro Gomes B. (2022). Nurses’ performance in palliative care: Spiritual care in the light of Theory of Human Caring. Rev. Bras. Enferm..

[22] Attum B., Hafiz S., Malik A., Shamoon Z. (2024). Cultural Competence in the Care of Muslim Patients and Their Families.

[23] Dos Santos F.C., Macieira T.G.R., Yao Y., Hunter S., Madandola O.O., Cho H., Bjarnadottir R.I., Lopez K.D., Wilkie D.J., Keenan G.M. (2022). Spiritual Interventions Delivered by Nurses to Address Patients’ Needs in Hospitals or Long-Term Care Facilities: A Systematic Review. J. Palliat. Med..

[24] Voetmann S.S., Hvidt N.C., Viftrup D.T. (2022). Verbalizing spiritual needs in palliative care: A qualitative interview study on verbal and non-verbal communication in two Danish hospices. BMC Palliat. Care.

[25] Liefbroer A.I., Ganzevoort R.R., Olsman E. (2019). Addressing the spiritual domain in a plural society: What is the best mode of integrating spiritual care into healthcare?. Ment. Health Relig. Cult..

[26] Heidari A., Afzoon Z., Heidari M. (2022). The correlation between spiritual care competence and spiritual health among Iranian nurses. BMC Nurs..

[27] Seid K., Abdo A. (2022). Nurse’s spiritual care competence in Ethiopia: A multicenter cross-sectional study. PLoS ONE.

[28] Wang Z., Zhao H., Zhang S., Wang Y., Zhang Y., Wang Z., Li X., Xiao L., Zhu Y., Han G. (2022). Correlations among spiritual care competence, spiritual care perceptions and spiritual health of Chinese nurses: A cross-sectional correlational study. Palliat. Support. Care.

[29] Watson J. Watson Caring Science Institute (2024). Watson’s Caring Science & Human Caring Theory. https://www.watsoncaringscience.org/jean-bio/caring-science-theory/.

[30] Anandarajah G., Craigie F., Hatch R., Kliewer S., Marchand L., King D., Hobbs R., Daaleman T.P. (2010). Toward Competency-Based Curricula in Patient-Centered Spiritual Care: Recommended Competencies for Family Medicine Resident Education. Acad. Med..

[31] Abusafia A.H., Khraisat A.M.S., Tableb O.K., Al-Mugheed K., Alabdullah A.A., Abdelaliem S.M.F. (2024). The impact of a nursing spiritual care module on nursing competence: An experimental design. BMC Palliat. Care.

[32] Babamohamadi H., Tafreshi A., Khoshbakht S., Ghorbani R., Asgari M.R. (2022). Nursing Students’ Professional Competence in Providing Spiritual Care in Iran. J. Relig. Health.

[33] Guo Z., Zhang Y., Li P., Zhang Q., Shi C. (2023). Student nurses’ spiritual care competence and attitude: An online survey. Nurs. Open.

[34] Karaca T., Altınbaş Y. (2024). Spiritual Care Support Perception and Spiritual Care Competence of Nursing Students in Turkey: A Quasi-Experimental Study. J. Relig. Health.

[35] Sarrión-Bravo J.A., González-Aguña A., Abengózar-Muela R., Renghea A., Fernández-Batalla M., Santamaría-García J.M., Ruiz-Moral R. (2022). Competence in Spiritual and Emotional Care: Learning Outcomes for the Evaluation of Nursing Students. Healthcare.

[36] Baldacchino D. (2015). Spiritual Care Education of Health Care Professionals. Religions.

[37] Laranjeira C., Benito E., Dixe M.A., Dones M., Specos M., Querido A. (2023). SPACEE Protocol: “Spiritual Care Competence” in PAlliative Care Education and PracticE: Mixed-Methods Research in the Development of Iberian Guidelines. Int. J. Environ. Res. Public Health.

[38] Tricco A.C., Lillie E., Zarin W., Brien K.K.O., Colquhoun H., Levac D., Moher D., Peters M.D.J., Horsley T., Weeks L. (2018). PRISMA Extension for Scoping Reviews (PRISMA-ScR): Checklist and Explanation. Ann. Intern. Med..

[39] Mak S., Thomas A. (2022). Steps for Conducting a Scoping Review. J. Grad. Med. Educ..

[40] Page M.J., McKenzie J.E., Bossuyt P.M., Boutron I., Hoffmann T.C., Mulrow C.D., Shamseer L., Tetzlaff J.M., Akl E.A., Brennan S.E. (2021). The PRISMA 2020 statement: An updated guideline for reporting systematic reviews. BMJ.

[41] Aromataris E., Munn Z. (2020). JBI Manual for Evidence Synthesis.

[42] Peters M., Godfrey C., McInerney P., Munn Z., Trico A., Khalil H. (2020). Chapter 11: Scoping Reviews. JBI Manual for Evidence Synthesis.

[43] Aromataris E., Lockwood C., Porritt K., Pilla B., Jordan Z. (2024). JBI Manual for Evidence Synthesis.

[44] Pollock D., Peters M.D.J., Khalil H., McInerney P., Alexander L., Tricco A.C., Evans C., de Moraes É.B., Godfrey C.M., Pieper D. (2023). Recommendations for the extraction, analysis, and presentation of results in scoping reviews. JBI Evid. Synth..

[45] Arksey H., O’Malley L. (2005). Scoping studies: Towards a methodological framework. Int. J. Soc. Res. Methodol..

[46] van Leeuwen R., Tiesinga L.J., Middel B., Post D., Jochemsen H. (2009). The validity and reliability of an instrument to assess nursing competencies in spiritual care. J. Clin. Nurs..

[47] Giske T., Schep-Akkerman A., Bø B., Cone P.H., Moene Kuven B., Mcsherry W., Owusu B., Ueland V., Lassche-Scheffer J., van Leeuwen R. (2022). Developing and testing the EPICC Spiritual Care Competency Self-Assessment Tool for student nurses and midwives. J. Clin. Nurs..

[48] Oliver A., Benito E., Sansó N., Galiana L. (2016). Cuestionarios de atención espiritual en cuidados paliativos: Revisión de la evidencia para su aplicación clínica. Psicooncologia.

[49] American Counseling Association Code of Ethics (2009). Competencies for Addressing Spiritual and Religious Issues in Counseling. https://www.counseling.org/docs/default-source/competencies/competencies-for-addressing-spiritual-and-religious-issues-in-counseling.pdf?sfvrsn=aad7c2c_12.

[50] Benito E., Dones M., Babero J. (2016). El acompañamiento espiritual en cuidados paliativos. Psicooncologia.

[51] Comprehensive Cancer Centres (IKNL) (2013). Spiritual Care. National Guideline. Version 1. https://www.sicp.it/wp-content/uploads/2018/12/2_Spiritualcare.pdf.

[52] Cone P., Giske T. (2022). Mental Health Staff Perspectives on Spiritual Care Competencies in Norway: A Pilot Study. Front. Psychol..

[53] Cooper D., Aherne M., Pereira J. (2010). The Competencies Required by Professional Hospice Palliative Care Spiritual Care Providers. J. Palliat. Med..

[54] DeFoor M.T., Moses M.M., Flowers W.J., Sams R.W. (2021). Medical student reflections: Chaplain shadowing as a model for compassionate care training. Med. Teach..

[55] Dezorzi L.W., Raymundo M.M., Goldim J.R., de Oliveira C.A.V. (2019). Spirituality in the continuing education of healthcare professionals: An approach to palliative care. Palliat. Support. Care.

[56] Hull C.E., Suarez E.C., Hartman D. (2016). Developing Spiritual Competencies in Counseling: A Guide for Supervisors. Couns. Values.

[57] Jurado S.R., Bassler T.C., Moreira A.S., Silva A.V., Dettmer S.A., Sanchez A. (2019). A espiritualidade e a enfermagem—Uma importante dimensão do cuidar. Rev. Nurs..

[58] Lazzaro C.V.B., Lucas C.B. (2022). Occupational Therapy’s Role in Understanding the Subjectivity of Spiritual Suffering. Occup. Ther. Ment. Health.

[59] Miner-Williams D. (2007). Connectedness in the Nurse-patient Relationship: A Grounded Theory Study. Issues Ment. Health Nurs..

[60] Mthembu T.G., Ahmed F., Nkuna T., Yaca K. (2015). Occupational Therapy Students’ Perceptions of Spirituality in Training. J. Relig. Health.

[61] Puchalski C., Ferrell B., Virani R., Otis-Green S., Baird P., Bull J., Chochinov H., Handzo G., Nelson-Becker H., Prince-Paul M. (2009). Improving the Quality of Spiritual Care as a Dimension of Palliative Care: The Report of the Consensus Conference. J. Palliat. Med..

[62] Rykkje L., Søvik M.B., Ross L., McSherry W., Cone P., Giske T. (2022). Educational interventions and strategies for spiritual care in nursing and healthcare students and staff: A scoping review. J. Clin. Nurs..

[63] UK Board of Chaplains (2020). Spiritual Care Competences for Healthcare Chaplains. United Kingdom. https://www.ukbhc.org.uk/wp-content/uploads/2020/10/UKBHC-CCs-180220.pdf.

[64] van Meurs J., Wichmann A.B., van Mierlo P., van Dongen R., van de Geer J., Vissers K., Leget C., Engels Y. (2022). Identifying, exploring and integrating the spiritual dimension in proactive care planning: A mixed methods evaluation of a communication training intervention for multidisciplinary palliative care teams. Palliat. Med..

[65] Vieten C., Scammell S., Pierce A., Pilato R., Ammondson I., Pargament K.I., Lukoff D. (2016). Competencies for psychologists in the domains of religion and spirituality. Spiritual. Clin. Pract..

[66] Batstone E., Bailey C., Hallett N. (2020). Spiritual care provision to end-of-life patients: A systematic literature review. J. Clin. Nurs..

[67] Lukovsky J., McGrath E., Sun C., Frankl D., Beauchesne M.A. (2021). A Survey of Hospice and Palliative Care Nurses’ and Holistic Nurses’ Perceptions of Spirituality and Spiritual Care. J. Hosp. Palliat. Nurs..

[68] van Leeuwen R., Tiesinga L.J., Middel B., Post D., Jochemsen H. (2008). The effectiveness of an educational programme for nursing students on developing competence in the provision of spiritual care. J. Clin. Nurs..

[69] Mächler R., Straßner C., Sturm N., Krisam J., Stolz R., Schalhorn F., Valentini J., Frick E. (2023). GPs’ Personal Spirituality, Their Attitude and Spiritual Competence: A Cross-Sectional Study in German General Practices. J. Relig. Health.

[70] Elias A.C.A., Giglio J.S., Pimenta C.A.d.M., El-Dash L.G. (2007). Development of a Brief Psychotherapy modality entitled RIME in a hospital setting using alchemical images. Arch. Clin. Psychiatry.

[71] Machul M., van Leeuwen R., Ozga D., Jurek K., Boczkowska S., Dobrowolska B. (2022). The level of spiritual care competence of Polish nurses and the psychometric properties of the spiritual care competence scale (SCCS). BMC Nurs..

[72] van de Geer J., Groot M., Andela R., Leget C., Prins J., Vissers K., Zock H. (2017). Training hospital staff on spiritual care in palliative care influences patient-reported outcomes: Results of a quasi-experimental study. Palliat. Med..

[73] Jafari M., Fallahi-Khoshknab M. (2021). Competence in providing spiritual care and its relationship with spiritual well-being among Iranian nurses. J. Educ. Health Promot..

[74] Rivas Chapoñan J., Cervera Vallejos M., Díaz Manchay R. (2022). Intervención terapéutica trascendental del profesional de enfermería al familiar acompañante en etapa de duelo. Rev. Cubana Enferm.

[75] Paul R., Elder L. (2005). The Miniature Guide to Understanding the Foundations of Ethical Reasoning. https://www.criticalthinking.org/files/SAM-EthicalReasoning2005.pdf.

[76] Benner P.E. (2001). From Novice to Expert: Excellence and Power in Clinical Nursing Practice.

[77] Baldacchino D. (2016). ‘Being in Doing’: ‘MERVIC’ Characteristics for Nurses’ Delivery of Spiritual Care. Ann. Nurs. Pract..

[78] Wolfteich C.E., Sandage S.J., Tomlinson J., Mettasophia J., Ventura D. (2019). Humility and Spirituality: New Directions in Interdisciplinary Research. Spirit. A J. Christ. Spiritual..

[79] Wright J.C., Nadelhoffer T., Perini T., Langville A., Echols M., Venezia K. (2017). The psychological significance of humility. J. Posit. Psychol..

[80] Gibbs G. (2001). Learning by Doing: A Guide to Teaching and Learning Methods. Geography Discipline Network.

[81] Schön D. (1992). La Formación de Profesionales Reflexivos. Hacia un Nuevo Diseño de la Enseñanza y el Aprendizaje en las Profesiones [A Formação de Profissionais Reflexivos. Rumo a uma Nova Concepção de Ensino e Aprendizagem nas Profissões].

[82] Drigas A., Mitsea E. (2020). The Triangle of Spiritual Intelligence, Metacognition and Consciousness. Int. J. Recent Contrib. Eng. Sci. IT.

[83] Jones K.F., Paal P., Symons X., Best M.C. (2021). The Content, Teaching Methods and Effectiveness of Spiritual Care Training for Healthcare Professionals: A Mixed-Methods Systematic Review. J. Pain Symptom Manag..

[84] Ahmadi M., Estebsari F., Poormansouri S., Jahani S., Sedighie L. (2021). Perceived professional competence in spiritual care and predictive role of spiritual intelligence in Iranian nursing students. Nurse Educ. Pract..

[85] Salman M., Ganie S., Saleem S. (2020). The concept of competence: A thematic review and discussion. Eur. J. Train. Dev..

[86] Cao Y., Kunaviktikul W., Petrini M., Sripusanapan A. (2020). Proposing a conceptual framework of spiritual care competence for Chinese nurses. Nurs. Health Sci..

[87] Pinto C.T., Veiga F., Guedes L., Pinto S., Nunes R. (2023). Models of spiritual intelligence interventions: A scoping review. Nurse Educ. Pract..

[88] Manso D., Capelas M.L. (2021). Translation and cross-cultural adaptation to European Portuguese of the Cuestionario GES (SECPAL). Cad. Saúde.

[89] Hsieh S.I., Hsu L.L., Hinderer K.A., Lin H.L., Tseng Y.P., Kao C.Y., Lee C.-Y., Kao S.-H., Chou Y.-F., Szu L.-Y. (2022). The Effects of a Scenario-Based Spiritual Care Course on Spiritual Care Competence among Clinical Nurses: A Quasi-Experimental Study. Healthcare.

[90] Shamsi M., Khoshnood Z., Farokhzadian J. (2022). Improving psychiatric nurses’ competencies in spiritual care and integration of clients’ religion/spirituality into mental healthcare: Outcomes of an online spiritual care training program. BMC Psychiatry.

[91] Vitorino L.M., Machado Teixeira P.H., Dominato P.C., de Azevedo M.P.C., Resende M.M., Lucchetti G. (2024). The effectiveness of spiritual care training on medical students’ self-reported competencies: A quasi-experimental study. Palliat. Support. Care.

[92] Pan C.X., Spinelli A., Litrivis E., Popoviciu A., Thomson K.P., Brondolo E. (2023). AD-LAST! An interdisciplinary clinical workshop to improve cultural and spiritual awareness in advance care planning skills. Palliat. Support. Care.

[93] Bolarinwa F.I., Esan D.T., Bolarinwa O.A. (2023). Assessment of Spiritual Care Practices among Nurses Caring for Cancer Patients in a Tertiary Hospital in Nigeria. SAGE Open Nurs..

[94] Afonso A., Sitefane S., Rabiais I., Nunes L., Caldeira S. (2023). Spiritual Care in the Undergraduate Nursing Degree in Portugal. Educ. Sci..

[95] Kurtgöz A., Edis E.K. (2023). Spiritual care from the perspective of family caregivers and nurses in palliative care: A qualitative study. BMC Palliat. Care.

[96] Goswami D.C. (2013). Understanding Self-Awareness in Palliative Care. J. Pain Palliat. Care Pharmacother..

[97] López-Tarrida A.C., Ruiz-Romero V., González-Martín T. (2020). Cuidando con sentido: La atención de lo espiritual en la práctica clínica desde la perspectiva del profesional. Rev. Esp. Salud Públic.

[98] Ventura F., Costeira C.R.B., Silva R., Cardoso D., Oliveira C. (2022). Person-Centered Practice in the Portuguese Healthcare Services: A Scoping Review Protocol. Nurs. Rep..

[99] Pentaris P., Tripathi K. (2022). Palliative Professionals’ Views on the Importance of Religion, Belief, and Spiritual Identities toward the End of Life. Int. J. Environ. Res. Public Health.

[100] Clyne B., O’Neill S.M., Nuzum D., O’Neill M., Larkin J., Ryan M., Smith S.M. (2022). Patients’ spirituality perspectives at the end of life: A qualitative evidence synthesis. BMJ Support Palliat. Care.

[101] Murgia C., Notarnicola I., Caruso R., De Maria M., Rocco G., Stievano A. (2022). Spirituality and Religious Diversity in Nursing: A Scoping Review. Healthcare.

