# Supplementary material for: Spiritual Care[Givers] Competence in Palliative Care: A Scoping Review
Source: Healthcare (Basel). 2024 May 22;12(11):1059. doi: 10.3390/healthcare12111059 (PMC11171750; doi:10.3390/healthcare12111059)
Supplement: Supplementary file 1 [file healthcare-12-01059-s001.zip › Supplementary File S1_Spiritual competence.pdf]

Table S1. Summary of the included studies (n = 30).

(Authors, year)	Title	Country	Type of Study	Objective(s)	Data Collection / Sample/ Setting	Main Findings
(American Counseling Association, 2009) [49]	Competencies for Addressing Spiritual and Religious Issues in Counseling.	USA	Guidelines	To identify competencies for Addressing Spiritual and Religious Issues in Counseling	N/A	"These competencies are intended to be used in conjunction with counseling approaches that are evidence-based and align with best practices in counseling" (p.1).<br>These were identified as spiritual competencies: Culture and Worldview; Counselor Self-Awareness; Human and Spiritual Development; Communication; Assessment; Diagnosis and Treatment.
(Baldacchino , 2015) [36]	Spiritual Care Education of Health Care Professionals.	Malta	Review	"Present the theories and methods of clinical education on spiritual care of health care professionals and students and outline the dimensions of spiritual leadership to sustain the learning process" (p.596).	Using theoretical models and recommendations to present theories and methods of clinical education on the spiritual care of health care professionals and students.	"The essence of spiritual care is being in doing, whereby personal spirituality and therapeutic use of oneself contribute towards effective holistic care. While taking into consideration the factors that may inhibit and enhance the delivery of spiritual care, recommendations are proposed for the educational, clinical, and management sectors for further research, and personal spirituality is recommended to ameliorate patient holistic care" (p.594).
(Batstone & Hallet, 2020) [66]	Spiritual care provision to end-of-life patients: A systematic literature review.	USA	Review	"Develop an understanding of how nurses provide spiritual care to terminally ill adult patients, when spiritual need is potentially the greatest, by identifying the literature on nurses' experiences of providing spiritual care" (p.3611).	Deductive thematic analysis. Nineteen electronic databases were systematically searched, and papers were screened (about nurses who care for end-of-life patients).	"Eleven studies provided a tripartite understanding of spiritual caregiving within the a priori themes: Nursing Spirit (a spiritual holistic ethos); the Soul of Care (the nurse–patient relationship); and the Body of Care (nurse care delivery). Ten studies involved PC nurses. Nurses who provide spiritual care operate from an integrated holistic worldview, which develops from personal spirituality, life experience, and professional practice of working with the dying. This worldview, when combined with advanced communication skills, shapes a relational way of spiritual caregiving that extends warmth, love, and acceptance, thus enabling a patient's spiritual needs to surface and be resolved" (p.3609).
(Benito et al., 2016) [50]	El acompañamiento espiritual en cuidados paliativos.	Spain	Review	Explore synthetically what is understood by spirituality, clinical spirituality, spiritual resources, and needs, the journey of a person going through a suffering, loss, or dying process, and the attitudes and tools of professionals to explore and accompany this difficult path.	Using theoretical guidelines and spiritual research groups to explore and synthesize information.	Spirituality is an important dimension in PC. Spiritual healing depends partly on the maturity and experience of the healer, since no one can accompany another along their journey. Competence in spiritual care can be developed. Moments of crisis are opportunities for spiritual emergency, they create an opportunity to encounter oneself, others, and that which transcends us, allowing us to experience what we are at our core.
(Comprehensive Cancer Centres	Spiritual care.	Netherlands	Guidelines	Determine appropriate care and treatment (p.2).	N/A	"Pay attention to the spiritual process from the very beginning of the palliative phase" (p.4); "develop sensitivity for detecting signals on spiritual issues, as these are often not

(Authors, year)	Title	Country	Type of Study	Objective(s)	Data Collection / Sample/ Setting	Main Findings
(IKNL), 2013) [51]						immediately apparent" (p.4); and "develop a 'refraining mode" (p.5).
(Cone & Giske, 2022) [52]	Mental Health Staff Perspectives on Spiritual Care Competencies in Norway: A Pilot Study.	Norway	Mixed-methods study	"Evaluate use of the tool among mental health staff, and secondly describe the views on spirituality and spiritual care of healthcare personnel working in a Norwegian mental health institution and identify their knowledge, skills, and attitudes related to spirituality and spiritual care of patients in their workplace" (p.2).	Survey of healthcare personnel (n = 24) from different professional backgrounds (nurse, social educator, nurse assistants, aides) (convenience sample).	Most of the staff indicated a lack of training related to the spiritual domain. "Awareness about spiritual care and especially regarding how to develop discernment of how and when to talk with patients in ways that could be supportive, even when patients are delusional or in crisis" (p.7).
(Cooper et al., 2010) [53]	The competencies required by professional hospice PC spiritual care providers.	Canada	Case Study	"Describe a Canadian Community of Practice process to develop an occupational analysis-based competency profile for the Professional Hospice PC Spiritual Care Provider. Developing a Curriculum (DACUM) methodology" (p.869).	"Conducted a preliminary informal analysis of the status of Hospice PC concerning the spiritual care developed in Canada" (p.870).	"Competency profiles are important contributions to the development of curricula to train care providers who are recognized by other professions and by institutions as possessing the requisite theoretical and clinical expertise, particularly in academic tertiary care settings" (p.869).
(DeFoor et al., 2021) [54]	Medical student reflections: Chaplain shadowing as a model for compassionate care training.	USA	Qualitative study	"Explore perceived benefits among medical students of pastoral care shadowing in integrating compassion and spirituality into education curricula" (p.101).	Written reflections from first- and second-year medical students (n = 64) were collected and analyzed, from December 2018 to January 2020 after shadowing with hospital chaplains.	"Four major themes were identified (1) learned values within pastoral care, (2) learned roles of pastoral care in the healthcare setting, (3) practiced spiritual assessment tools and resource identification, and (4) reflected personal impact on future career" (p.101).
(Dezorzi, et al., 2019) [55]	Spirituality in the continuing education of healthcare professionals: An approach to PC.	Brazil	Quantitative study	"Evaluate the effectiveness of a continuing education activity for healthcare professionals on spirituality and spiritual care for patients/families in PC at a public hospital in southern Brazil" (p.2).	The Brazilian version of the Spiritual Care Competence Scale was applied before and after attending a four-hour continuing education activity with healthcare professionals (n = 52 in first meeting and n = 42 in second meeting).	"Significant differences were observed between pre- and post-intervention scores in the following dimensions: assessment and implementation of spiritual care; professionalization and improving the quality of spiritual care; personal support; patient counseling; and referral" (p.1).
(Elias et al, 2017) [70]	Development of a Brief Psychotherapy modality entitled RIME in a hospital setting using alchemical images	Brazil	Qualitative study	"Analyze a training program by understanding the experience of professionals in using the RIME (relaxing, mental images, and spirituality) Intervention and understanding the experience of patients in re-signifying spiritual	Collected 11 structured questionnaires, 21 semi-structured interviews, and 11 diaries. The sample was a nurse, a doctor, three psychologists, and a volunteer alternative therapist, all experienced or experts in PC,	"In analyzing the professionals' experience, five categories and 15 subcategories were found" (p.61). In analyzing the nature of spiritual pain, the most prevalent categories were fear of death (expressed through denial) and perception of the clinical picture. When applying RIME, there was a statistically significant difference: at the end of the session, patients

(Authors, year)	Title	Country	Type of Study	Objective(s)	Data Collection / Sample/ Setting	Main Findings
				pain, manifested during the application of RIME by trained professionals" (p.60).	selected by invitation, and who cared for 11 terminal patients admitted to public hospitals in Brazil.	reported a higher level of well-being than at the beginning of the session.
(Evangelista et al., 2021) [21]	Nurses' performance in PC: spiritual care in the light of Theory of Human Caring.	Brazil	Qualitative study	"Analyze nurses' role in assisting patients in PC, with emphasis on the spiritual dimension, in the light of the Theory of Human Caring" (p.1).	Semi-structured interviews with nurses (n = 10) assisting patients in PC.	"The spiritual dimension of care is contemplated by several religious and spiritual practices. These are respected and encouraged by nurses, although there is difficulty in providing care for the spiritual dimension. Nurses have attitudes consistent with Jean Watson's Theory and apply the Caritas Process elements during assistance to patients' spiritual dimension in PC" (p.1).
(Heidari et al., 2022) [26]	The correlation between spiritual care competence and spiritual health among Iranian nurses.	Iran	Quantitative study	"Investigate correlation between the spiritual health and spiritual care competence of Iranian nurses" (p.3-4).	Persian versions of the Spiritual Health Questionnaire (Amiri) and the Spiritual Care Competence Scale (SCCS). Study with Hospital Iranian nurses (n = 172).	"The study revealed that spiritual care competence of nurses is correlated with their spiritual health and performance, as a subscale of spiritual health can predict their spiritual care competence. Thus, it can be concluded that the spiritual health of nurses is an important factor in providing spiritual care for patients and meeting their spiritual needs" (p.1).
(Hull et al., 2016) [56]	Developing Spiritual Competencies in Counseling: A Guide for Supervisors.	USA	Guidelines	"Provide guidelines for counselors to implement in conjunction with evidence-based counseling practices" (p.111).	N/A	This article "describes specific supervision tools meant to develop ethical awareness and increase overall clinical competency related to addressing spirituality in counseling" (p.112). Competences: Culture and Worldview; Counselor Self-Awareness; Human and Spiritual Development; Communication; Assessment; Diagnosis and Treatment.
(Jafari & Fallahi-Khoshknab, 2021) [73]	Competence in providing spiritual care and its relationship with spiritual well-being among Iranian nurses.	Iran	Quantitative study	"Evaluate Iranian nurses' competence in providing spiritual care and its relationship with their Spiritual Well-Being" (p. 2).	A questionnaire with the Spiritual Care Competence Scale (SCCS) and the Spiritual Well-Being Scale (SWBS) was applied to Iranian nurses (n = 158).<br>All invited nurses were working in teaching hospitals affiliated with Bam University of Medical Sciences in the south of Iran between 2016 and 2017.	"The results showed that the mean scores of SCCS and SWBS were 101±12.6 and 76.92±13.4, respectively. Pearson correlation test showed a significant and direct relationship between the mean score of SCCS and SWBS. This test also showed a significant relationship between all the dimensions of SCCS and SWBS ($p<0.05$). Nurses had a relatively appropriate competence in providing spiritual care to patients. There was also a significant relationship between the nurses' spiritual care competency and their spiritual well-being" (p.1).
(Jurado et al., 2019) [57]	A espiritualidade e a enfermagem – Uma importante dimensão do cuidar [Spirituality and nursing – an important dimension of care]	Brazil	Review	"Identify forms of spiritual interventions during nursing care and the benefits of spirituality for patients" (p.3447).	Two databases were used: *Literatura Latino-Americana e do Caribe em Ciências da Saúde* (LILA-CS) and Scientific Electronic Library Online (SciELO) (n = 31 articles).	Despite the growing scientific production on this topic and the countless benefits of spiritual care for patients, the spiritual aspect requires greater attention from health professionals, especially nursing professionals, making it necessary to qualify them to better deal with this dimension, to promote holistic and comprehensive patient care.

(Authors, year)	Title	Country	Type of Study	Objective(s)	Data Collection / Sample/ Setting	Main Findings
(Lazzaro & Lucas. 2022) [58]	Occupational Therapy's Role in Understanding the Subjectivity of Spiritual Suffering.	USA	Narrative study	"Inform occupational therapy practitioners about the concept of spiritual suffering and reaffirm a focus on spirituality" (p.151).	"Expands on the construct of spirituality as defined in the Occupational Therapy Practice Framework (4th ed.) and provides a lens for knowledge translation to assist practitioners in narrative assessment and mindful treatment regarding spiritual suffering" (p.151).	"Through an understanding of existential and postmodern philosophy, practitioners may address the lived experience of spiritual suffering that may go unnoticed due to time, knowledge, understanding, and the social-cultural factors that affect service delivery" (P.151).
(Lukovsky et al., 2021) [67]	A Survey of Hospice and PC Nurses' and Holistic Nurses' Perceptions of Spirituality and Spiritual Care.	USA	Quantitative study	"Assess hospice and palliative nurses' and holistic nurses' perceptions of spirituality and spiritual care" (p.28).	Web-based survey to measure perception of spirituality and spiritual care provision using a modified Spirituality and Spiritual Care Rating Scale with palliative nurses and holistic nurses (n = 250).	"This study found that given adequate resources and education, nurses can be positioned to address the spiritual needs of patients and provide appropriate care. This study adds to an emerging body of evidence suggesting that training in spiritual care should be an important component of the foundational nursing curriculum" (p.28).
(Mächler et al., 2023) [69]	GPs´ Personal Spirituality, Their Attitude, and Spiritual Competence: A Cross-Sectional Study in German General Practices.	Germany	Quantitative study	"Understand whether there is an interrelation between spiritual competence, personal spirituality, and attitude towards enquiring about spirituality among general practitioners (GP)" (p.2436).	Spiritual Care Competence was measured using the Spiritual Care Competence Questionnaire (SCCQ) with German general practitioners (n = 30).	"Found correlations between GPs' personal spirituality, their spiritual competence, and their attitudes toward Spiritual Care (SC). The ability to perceive the spiritual needs of patients was the competence most strongly related to GPs' attitude towards SC. The competence with the strongest correlation to personal spirituality was Self-awareness and Proactive opening. The results show that GPs' personal spirituality and spiritual competence are indeed related to addressing spirituality with their patients. To foster Spiritual Care, training programs should raise awareness for one's personal spirituality and encourage one to reflect on spiritual competence" (p.2436).
(Machul et al, 2022) [71]	The level of spiritual care competence of Polish nurses and the psychometric properties of the spiritual care competence scale (SCCS).	Poland	Quantitative study	"Analyze the psychometric properties of the SCCS.<br>Analyze the level of spiritual competence of professionally active nurses in Poland" (p.1).	The following tools were used with Polish nurses (n = 343): Spiritual Care Competence Scale (SCCS), as developed by van Leeuwen et al.; the Duke University Religion Index (DUREL) in its Polish version; a short form collecting sociodemographic characteristics.	"The findings highlight the importance of providing professional education in spiritual nursing care, especially in its practical dimension developing skills in which nurses obtained lower scores" (p.1). Job seniority appears to be one of the main factors determining the level of competence in the provision of spiritual care.

(Authors, year)	Title	Country	Type of Study	Objective(s)	Data Collection / Sample/ Setting	Main Findings
(Miner-Williams. 2007) [59]	Connectedness in the nurse-patient relationship: a grounded theory study.	USA	Qualitative study	"Explore and describe Nurse-Patient Relationship Connectedness from the perspective of nurses and patients" (p.1216).	Fifteen people were interviewed, sharing 25 different stories of meaningful experiences from the nurse, patient, and family member perspectives.	"Findings highlighted that the nurse-patient relationship exists for the nurse to meet the health needs of the patient. Ordinarily, these are biopsychosocial needs. However, at times patients present needs emanating from deep within the person, which are deemed needs of the spirit. Under certain conditions, with a nurse who is competent and willing, a process evolves marked by meaningfulness, which not only meets these needs of the spirit but strongly impacts the nurse, the patient, or both, and promotes healing, growth, and comfort" (p.1215).
(Mthembu et al., 2015) [60]	Occupational Therapy Students' Perceptions of Spirituality in Training.	South Africa	Qualitative study	"Explore the occupational therapy students' perceptions of spirituality in training" (p.2178).	Four semi-structured interviews were conducted with students (n = 2), a lecturer (n = 1), and an occupational therapist (n = 1). In addition, two focus groups were conducted with students to collect data.	"The analysis resulted in four themes: "Unique to every individual," "Spirituality in occupational therapy," "To be or not to be taught," and "The Real world." Participants perceived spirituality as an individual experience" (p.2178).
(Puchalski et al., 2009) [61]	Improving the Quality of Spiritual Care as a Dimension of PC: The Report of the Consensus Conference.	USA	Consensus Report (Review)	"Identify points of agreement about spirituality as it applies to healthcare and make recommendations to advance the delivery of quality spiritual care in PC" (p.885).	"Five literature-based categories of spiritual care were defined: spiritual assessment, models of care and care plans, interprofessional team training, quality improvement, and personal and professional development. The document was built upon prior literature, the "NCP Guidelines 21" and the National Quality Forum (NQF) Preferred Practices and Conference proceedings" (p.885-886). The consensus was reached by a group including 40 representative national leaders: physicians, nurses, psychologists, social workers, chaplains, clergy, other spiritual care providers, and health care administrators. A panel of 150 expert reviewers provided additional comments. All participants of the Consensus Conference and the six project	"Studies have indicated the strong desire of patients with serious illness and end-of-life concerns to have spirituality included in their care. There is a strong empirical and scholarly body of literature to support the inclusion of spiritual care as part of a biopsychosocial–spiritual approach to care. Recommendations were made to improve the spiritual care provision" (p.902).

(Authors, year)	Title	Country	Type of Study	Objective(s)	Data Collection / Sample/ Setting	Main Findings
					advisors reviewed that Consensus Report had agreed to its content.	
(Rivas, et al., 2022) [74]	Intervención terapéutica trascendental del profesional de enfermería al familiar acompañante en etapa de duelo.	Peru	Qualitative Study	"Describe the transcendental therapeutic intervention of nursing professionals and provide support to the accompanying family member in the bereavement stage in a public hospital" (p.2).	Semi-structured interviews via Zoom and telephone call with PC nurses (n = 10) from a public hospital.	"Three categories emerged: 1) transcendental therapeutic interventions: farewell, condolences, accompaniment, and spiritual support to the accompanying family member in the bereavement stage; 2) influence of therapeutic interventions: acceptance and posttraumatic stress reduction in the face of bereavement; and 3) need for continuous education for a more effective intervention during bereavement" (p.2).
(Rykkje et al., 2021) [62]	Educational interventions and strategies for spiritual care in nursing and healthcare students and staff: A scoping review.	Norway	Review	"Map existing evidence about educational interventions or strategies in nursing and allied healthcare concerning students' and staff's spiritual care provision" (p.1440).	This study of 36 sources focuses upon spiritual care competencies for healthcare students and staff, with the primary focus upon nursing, and was guided by evidence-based methods for scoping reviews, namely the PRISMA (Preferred Reporting Items for Systematic Reviews and Meta-Analyses) extension for scoping reviews (PRISMA-ScR) checklist.	This review found a wide range of studies with insights into educational interventions or strategies for teaching nursing and allied healthcare students and staff about spiritual care. "The findings support the inclusion of spiritual care both in monodisciplinary and multidisciplinary educational settings, although there is no 'right' way or best standard to guide spiritual care curricula" (p.1440-1441).
(Seid & Abdo, 2022) [27]	Nurse's spiritual care competence in Ethiopia: A multicenter cross-sectional study.	Ethiopia	Quantitative study	"Evaluate the current state of spiritual care competence and the factors that influence it among nurses in Southwest Ethiopia" (p.1).	The Spiritual Care Competence Scale (SCCS) was used to gather data on nurses' competence in spiritual care, in Southwest Ethiopia (n = 367).	"The mean spiritual care competence score among healthcare professionals was 3.14±0.74. Age ($p<0.05$) and training in spiritual care ($p<0.05$) were significantly associated with spiritual care competence" (p.1).
(UK Board of Chaplains, 2020) [63]	Spiritual Care Competences for Healthcare Chaplains.	UK	Guideline	"Detail the competences required of a UK Board of Healthcare Chaplains (p.2).	N/A	Chaplains continually develop and update their knowledge of spiritual and religious care, current policy, and evidence relevant to spiritual care services, which they use to promote and develop safe, effective, evidence-based practice.
(van Leeuwen, et al., 2008) [68]	The effectiveness of an educational program for nursing students on developing competence in the provision of spiritual care.	Netherlands	Quantitative study	"Determine the effects of a course for nursing students on developing competence in spiritual care and the factors that might influence the effects (p.2768).	Questionnaire that covered all the main nursing competencies generally expected to be present in advanced-beginner nurses in the Netherlands from Christian nursing schools (n = 97). The competences of spiritual care were measured with the Spiritual Care Competence Scale (SCCS).	"Statistically significant changes in scores on three subscales of the Spiritual Care Competence Scale between groups (T1) and over time for the whole cohort of students on all subscales (T2). Clinical placement was a negative predictor of three subscales of the SCCS. Experience in spiritual care and a holistic vision of nursing were both positive predictors of certain competencies. A statistically significant difference was observed between groups in the student analysis of a vignette with explicit spiritual content" (p.2768).

(Authors, year)	Title	Country	Type of Study	Objective(s)	Data Collection / Sample/ Setting	Main Findings
(van Meurs et al., 2022) [64]	Identifying, exploring and integrating the spiritual dimension in proactive care planning: A mixed methods evaluation of a communication training intervention for multidisciplinary PC teams.	Netherlands	Mixed-methods study	"Evaluate the effects of an interactive communication training intervention for PC teams to identify and explore the spiritual dimension and integrate them in patient care plans" (p.1493).	Self-assessment questionnaires, evaluation of videos with simulated consultations (applied competence) and medical record review (implementation). Three types of PC teams (primary and secondary): nurses (n = 21), physicians (n = 14) and spiritual caregivers (n = 3).	Training intervention resulted in increased PC professionals' competence in identifying and exploring patients' spiritual issues, and their integration into multidimensional proactive PC plans. "The intervention directly addresses patients' spiritual concerns and adds value to their PC plans" (p.1483).
(Vieten et al., 2016) [65]	Competencies for psychologists in the domains of religion and spirituality.	USA	Quantitative study	"Investigate how a more general sample of psychologists respond to the spiritual and religious competencies" (p.96).	A sample of psychologists (n = 272) were asked to complete four ratings for each of the 16 proposed competencies.	Results of this survey study demonstrate a very large degree of support for the proposed competencies. More than 70% to 90% of "respondents agreed that psychologists should receive training and demonstrate competence in each of the 16 domains" (p.96). This overwhelming majority of positive responses may reflect psychologists' increasing recognition of spirituality and religion as important aspects of human diversity, as well as their importance in people's psychological lives.
(Zock et al., 2017) [72]	Training hospital staff on spiritual care in PC influences patient-reported outcomes.	Netherlands	Quantitative study	"Measure the effects of a specific spiritual care training on patients' reports of their perceived care and treatment" (p.743).	Questionnaires on physical symptoms, spiritual distress, involvement, attitudes (Spiritual Attitude and Involvement List), and the perceived focus of healthcare professionals on patients' spiritual needs. The following instruments were used: Dutch translation of the Supportive and PC Indicators Tool (OPZIS); Utrecht Symptom Diary; spiritual items adapted from the Distress Thermometer; items from the Spiritual Attitude and Interests; and six items related to Spiritual Care from the report on consumer quality indicators of PC. The intervention was a specific spiritual care training implemented by healthcare chaplains to eight multidisciplinary teams in six hospitals on regular wards.	"Patients (n = 85) had high scores on spiritual themes and involvement. Patients reported that attention to their spiritual needs was very important. There was a significant (p = 0.008) effect on healthcare professionals' attention to patients' spiritual and existential needs and a significant (p = 0.020) effect in favor of patients' sleep. No effect on the spiritual distress of patients or their proxies was found. The effects of spiritual care training can be measured using patient-reported outcomes and seemed to indicate a positive effect on the quality of care" (p.743).
